# Ultrasound-guided robotic surgical procedures: a systematic review

**DOI:** 10.1007/s00464-024-10772-4

**Published:** 2024-03-21

**Authors:** Matteo Pavone, Barbara Seeliger, Elena Teodorico, Marta Goglia, Cristina Taliento, Nicolò Bizzarri, Lise Lecointre, Cherif Akladios, Antonello Forgione, Giovanni Scambia, Jacques Marescaux, Antonia C. Testa, Denis Querleu

**Affiliations:** 1 Institute of Image-Guided Surgery, IHU Strasbourg, Strasbourg, France; 2 Research Institute Against Digestive Cancer, IRCAD, Strasbourg, France; 3 UOC Ginecologia Oncologica, Dipartimento di Scienze della Salute della Donna, del Bambino e di Sanità Pubblica, Fondazione Policlinico Universitario A. Gemelli, IRCCS, Rome, Italy; 4 Department of Digestive and Endocrine Surgery, University Hospitals of Strasbourg, Strasbourg, France; 5 ICube, UMR 7357 CNRS, University of Strasbourg, Strasbourg, France; 6 Department of Medical and Surgical Sciences and Translational Medicine, Faculty of Medicine and Psychology, Sapienza University of Rome, Rome, Italy; 7 Department of Obstetrics and Gynecology, University Hospital Ferrara, Ferrara, Italy; 8 Department of Obstetrics and Gynecology, University Hospitals Leuven, 3000 Leuven, Belgium; 9 Department of Gynecologic Surgery, University Hospitals of Strasbourg, Strasbourg, France

**Keywords:** Image-guided surgery, Robotic-assisted surgery, Ultrasound, Digital surgery, Artificial intelligence, New technologies

## Abstract

**Introduction:**

Ultrasound has been nicknamed “the surgeon’s stethoscope”. The advantages of laparoscopic ultrasound beyond a substitute for the sense of touch are considerable, especially for robotic surgery. Being able to see through parenchyma and into vascular structures enables to avoid unnecessary dissection by providing a thorough assessment at every stage without the need for contrast media or ionising radiation. The limitations of restricted angulation and access within the abdominal cavity during laparoscopy can be overcome by robotic handling of miniaturised ultrasound probes and the use of various and specific frequencies will meet tissue- and organ-specific characteristics. The aim of this systematic review was to assess the reported applications of intraoperative ultrasound-guided robotic surgery and to outline future perspectives.

**Methods:**

The study adhered to the PRISMA guidelines. PubMed, Google Scholar, ScienceDirect and ClinicalTrials.gov were searched up to October 2023. Manuscripts reporting data on ultrasound-guided robotic procedures were included in the qualitative analysis.

**Results:**

20 studies met the inclusion criteria. The majority (53%) were related to the field of general surgery during liver, pancreas, spleen, gallbladder/bile duct, vascular and rectal surgery. This was followed by other fields of oncological surgery (42%) including urology, lung surgery, and retroperitoneal lymphadenectomy for metastases. Among the studies, ten (53%) focused on locating tumoral lesions and defining resection margins, four (15%) were designed to test the feasibility of robotic ultrasound-guided surgery, while two (10.5%) aimed to compare robotic and laparoscopic ultrasound probes. Additionally two studies (10.5%) evaluated the robotic drop-in probe one (5%) assessed the hepatic tissue consistency and another one (5%) aimed to visualize the blood flow in the splenic artery.

**Conclusion:**

The advantages of robotic instrumentation, including ergonomics, dexterity, and precision of movements, are of relevance for robotic intraoperative ultrasound (RIOUS). The present systematic review demonstrates the virtue of RIOUS to support surgeons and potentially reduce minimally invasive procedure times.

In the last few decades, there has been a rapid succession of technological advances, marking a radical shift from the open to the minimally invasive surgical (MIS) approach [1]. The advantages of laparoscopy over laparotomy are now widely acknowledged [2]. Over the past twenty-five years, robotic surgery has experienced a raise and today, with the availability of several platforms alongside the continuously leading da Vinci systems (Intuitive Surgical Inc., Sunnyvale, CA, USA), robotic approaches are playing an increasingly crucial role [3, 4]. Despite observed advantages for certain patient characteristics (e.g., BMI > 30), challenges such as the lack of dedicated reimbursement, high costs and often longer operating times still limit the widespread use of robotic platforms worldwide [5]. Open surgery provides direct visual and tactile information of the explored regions. In contrast, MIS comes at the cost of predominantly two-dimensional view and limited tactile assessment. Intraoperative ultrasound (IOUS) is commonly utilized during open surgery with linear or finger probes, particularly in the hepatobiliary (HPB) and urological fields [6, 7]. In laparoscopic setting, ultrasound probes for guidance in MIS are more challenging to handle [8]. To overcome this limitation, innovative approaches for robotic platforms integrate ultrasound imaging to facilitate its use in MIS [9]. Image-guided robotic approaches, particularly those based on three-dimensional (3D) imaging, augmented reality (AR), and machine learning algorithms, offer advantages in the era of digital surgery [10]. Real-time, non-invasive, cost-effective and dynamic intraoperative imaging of complex anatomy are the main benefits of computer-assisted surgery. In this context, IOUS has emerged as the imaging modality of choice facilitated by the introduction of articulated robotic instruments to handle ultrasound probes [11]. The augmentation and fusion of imaging modalities are especially beneficial for delineating healthy and neoplastic tissue in oncological surgery [12]. The navigation of drop-in ultrasound probes manoeuvred by articulated robotic graspers provides access to anatomical spaces and angles that are inconvenient for relatively rigid laparoscopic probes. While initial reports of applications of intraoperative ultrasound during robotic surgery (RIOUS) have been published in the fields traditionally managed by open surgery, with encouraging results., pooled data are lacking [6, 13]. Therefore, the aim of this systematic review is to assess the reported applications of intraoperative ultrasound-guided robotic surgery and to outline future perspectives.

## Materials and methods

### Search strategy

The systematic review was conducted according to Preferred Reporting Items for Systematic Reviews and Meta-Analyses (PRISMA) guidelines [14] and registered with the International Prospective Register of Systematic Reviews PROSPERO (n CRD42023494430) prior to data extraction. Articles were obtained by querying the PubMed database, Google Scholar, ScienceDirect and ClinicalTrial.gov filtered by the English language up to October 2023 without additional restrictions. The database was retrieved through title and abstract screening using the following search terms: “intraoperative”, “robotic”, “surgery”, “ultrasound”, “laparoscopic”, “probe”.

### Data extraction

After removing duplicate publications, titles, abstracts, and keywords were independently reviewed by M.P and E.T. for inclusion, followed by full text review of eligible articles. In case of discrepancies, a consensus was reached through agreement with a third author (M.G.). The inclusion criterion was the description of ultrasound-assisted robotic surgical procedures. Excluded were articles without robotic use of the probe, as well as abstracts, reviews, meta-analyses, letters, and editorials. Studies reporting robotic ultrasound imaging independently of a surgical procedure, or those focused on percutaneous ultrasound-guided techniques and biopsies were also excluded. Data about the authors, surgical procedures, probes specifics and ultrasound-assisted robotic procedures were extracted for further analysis.

## Results

**Fig. 1:**
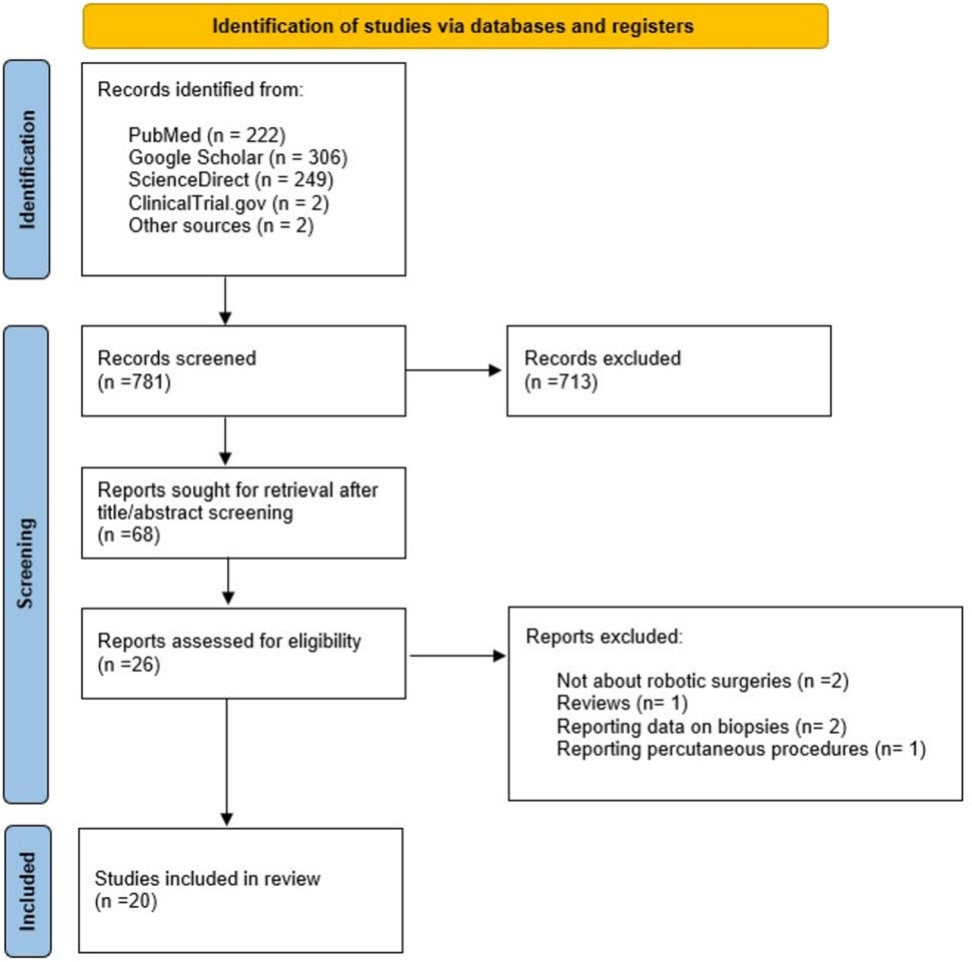
PRISMA flow diagram of study selection

The search strategy identified studies reporting intraoperative ultrasound imaging during robotic surgery. Initially, 781 studies were identified, and 68 full texts were selected through title and abstract screening. Finally, 20 studies met the inclusion criteria for the systematic review (Fig. 1). Due to the low number of reports, a qualitative analysis was performed [6–8, 11, 13, 15–28].

Among the included studies, two were prospective (10%), fifteen teen were retrospective (75%), three were experimental (15%), involving laboratory tests in vivo (porcine models) or on ex vivo phantoms. The studies were mainly (53%) from the field of general surgery during liver, pancreas, spleen, gallbladder/bile duct, vascular and rectal surgery [6, 8, 11, 16–22, 28]. The remaining studies(42%) covered other fields of oncological surgery including urology [7, 13, 23–26], lung surgery [27], and retroperitoneal lymphadenectomy for metastases [15].

Ten studies (53%) were focused on locating tumoral lesions and defining resection margins [7, 11, 13, 18–20, 22, 25, 27, 29]. Additionally, four studies (15%) were designed to assess the feasibility of robotic ultrasound-guided surgery [8, 15, 23], two (10.5%) aimed to compare robotic and laparoscopic ultrasound probes [6, 24], another two (10.5%) were conducted to evaluate the robotic drop-in probe [13, 16], one study (5%) focused on assessing hepatic tissue consistency [17] and another (5%) aimed to visualize the blood flow in the splenic artery [21].

**Fig. 2:**
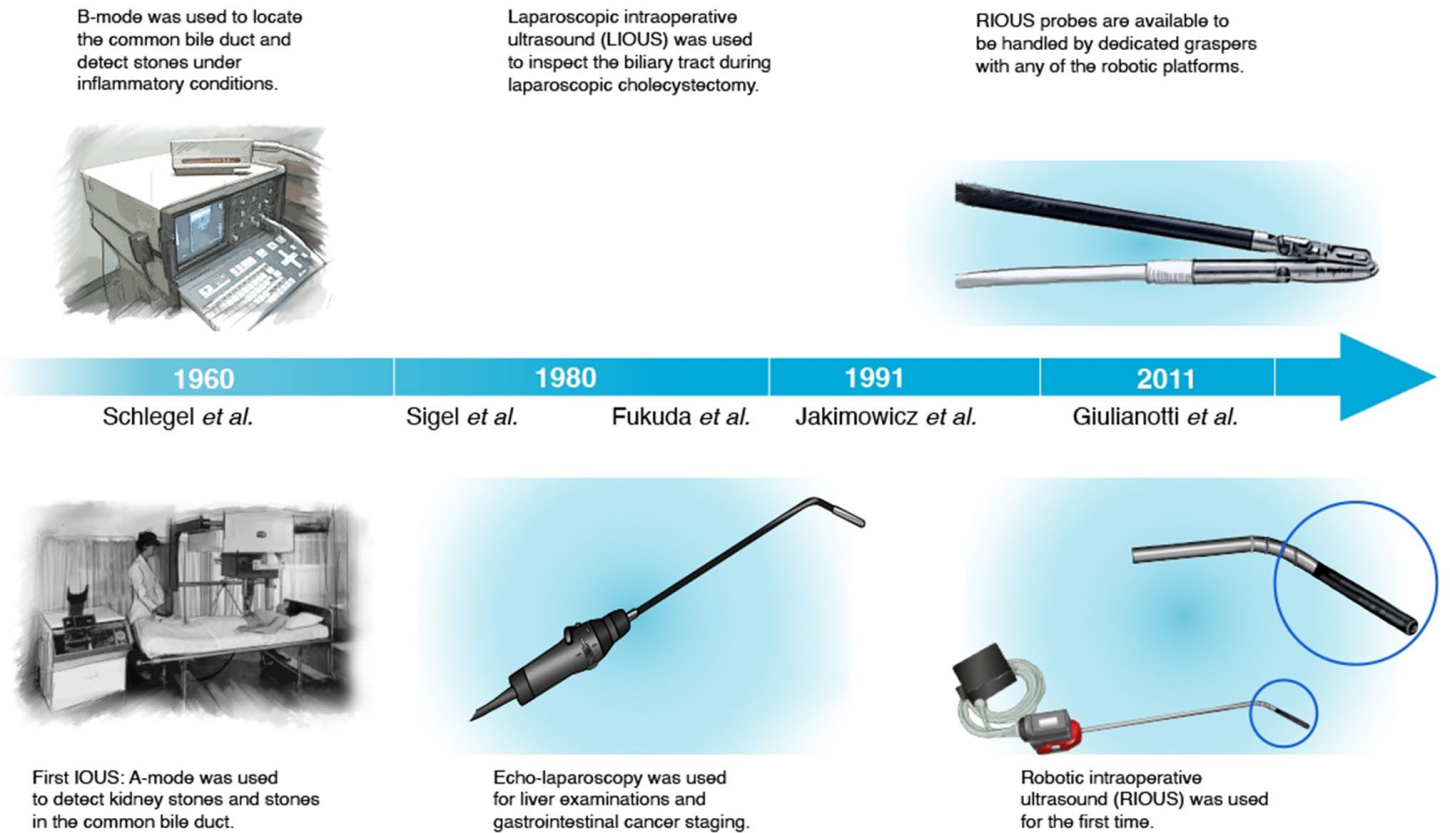
Timeline of intraoperative ultrasound techniques

In eleven articles (55%), a miniaturized linear drop-in probe was used [6, 7, 11, 13, 15, 16, 23–25, 27, 28]. These probes can be introduced via a 10–12 mm accessory trocar and steered from the surgeon’s console using robotic graspers (Fig. 2). Five manuscripts reported the use of rigid probes, which can be docked to the robotic arm (12 mm trocar) as prototypes corresponding to da Vinci robotic instruments (Fig. 2) [8, 21]. Alternatively, a laparoscopic articulated probe can be used during robotic surgery, introduced via the 10 mm accessory port and manipulated by the bedside assistant (Fig. 2). The ultrasound frequencies of the probes used in the included studies ranged from 3 to 13 MHz. All reported procedures were performed with the da Vinci robotic platforms. Table 1 summarizes the characteristics of the included articles, and details about ultrasound probes and surgical applications. No clinical trials on the use of RIOUS were registered at the timepoint of the database query. Due to the heterogeneity of data concerning probes, frequencies, procedures and study outcomes, a quantitative analysis of the results was deemed inappropriate.

**Table 1:** Study details and ultrasound

Year	Author	Country	Design	Sample Size	Ultrasound Target	Purpose	Probe docking	Probe Type	MHz	Platform
2011	Giulianotti et al. [1]	Italy	Case Series	6	Splenic Artery	Vascular blood flow	Robotic arm	NA	NA	Da Vinci
2012	Yakoubi et al. [2]	USA	Feasibility Test	In vivo porcine model	Kidney	To evaluate a novel ultrasound probe specifically developed for robotic surgery by determining its efficiency in identifying renal tumors	Robotic arm	BK Drop-In 8826	5–12 MHz	Da Vinci S
2012	Schneider et al. [3]	USA	Feasibility Test	In vivo animal model/Phantom Liver	Liver	In vivo porcine hepatic visualization and probe manipulation, lesion detection accuracy, and biopsy precision	Robotic arm	Rigid Robotic Laparoscopic (RLUS) probe	7.5 MHz	Da Vinci
2012	Kaczmarek et al. [4]	USA	Case Series	22	Kidney	Accurate tumor identification during partial nephrectomy	Robotic arm assisted	Dro-in Hitachi-Aloka	4–13 MHz	Da Vinci
2012	Billings et al. [5]	USA	Feasibility Test	Phantom tissue experiment	NA	Tissue hardness by elastography	Robotic arm	Rigid Robotic Laparoscopic (RLUS) probe	NA	Da Vinci Si
2013	Kaczmarek et al. [6]	USA	Retrospective	75	Kidney	To evaluate and compare perioperative outcomes of robotic partial nephrectomy (RPN) using robotic and laparoscopic ultrasound probe for tumor identification	Robotic arm	Drop-in Hitachi-Aloka	4–13 MHz	Da Vinci
2015	Pessaux et al. [7]	France	Case series	3	Liver	The explore the potential of AR navigation as a tool to improve safety of the surgical dissection is outlined for robotic hepatectomy	Robotic arm	NA	NA	Da Vinci
2015	Guerra et al. [8]	Italy	Case Series	10	Liver	To evaluate the feasibility and reliability of robotically integrated ultrasound to guide resection of malignant hepatic tumors	Robotic arm assisted	BK Drop-In 8826	5–12 MHz	Da Vinci Si
2015	Liu et al. [9]	China	Case Series	7	Pancreas	To seek for previously undetected lesions and to determine the accurate surgical resection margins in pancreatic tumors	Accessory trocar in robotic surgery	Rigid UST-5410 miniprobe (Aloka Alpha 7 Hitachi)	4–13 MHz	Da Vinci S
2016	Gunelli et al.[10]	Italy	Prospective	22	Kidney	Renal tumor enucleation	Robotic arm assisted	BK Drop-In 8826	5–12 MHz	Da Vinci
2017	Zhou et al. [11]	China	Prospective	17	Lung	To investigate the efficacy of intraoperative ultrasonographic localization during da Vinci thoracic surgery	Robotic arm assisted	BK Drop-In 8826	5–12 MHz	Da Vinci
2018	Araujo et al. [12]	Brazil	Case Series	2	Liver	Liver lesions identification	Robotic arm assisted	NA	NA	NA
2019	Zhang K et al. [13]	China	Case Series	1	Lymph node	To describe the robot-assisted laparoscopic management of post-chemotherapy retroperitoneal metastasis	Robotic arm assisted	BK Drop-In 8826	5–12 MHz	Da Vinci Si
2020	Sun et al. [14]	China	Retrospective	38	Kidney	To introduce the role and use of intraoperative ultrasound (IOUS) performed in robotic-assisted renal partial nephrectomy (RAPN) for endophytic renal tumors	Robotic arm assisted	BK Drop-In 8826	5–12 MHz	Da Vinci
2020	Chang et al. [15]	Taiwan	Prospective	93	Tongue	Explore soft laringeal tissues	Accessory trocar in Robotic surgery	Rigid UST-533 miniprobe (Aloka Alpha 7 Hitachi)	4–13 MHz	Da Vinci Si
2020	Zhang Y et al. [16]	China	Retrospective	29	Kidney	To report the experience in treating endophytic renal tumor by robot-assisted partial nephrectomy with a standard laparoscopic ultrasound probe	Accessory trocar in robotic surgery	Rigid UST-5550 (Hitachi Aloka Medical, Japan)	4–10 MHz	Da Vinci Si
2023	Di Mitri et al. [17]	Italy	Case Series	1	Spleen	Splenic cyst identification	Accessory trocar in Robotic surgery	2D rigid LPS US	NA	Da Vinci
2023	Glaysher et al. [18]	UK	Case Series	NA	Cholecystectomy	Delineate superiority of robotic-assisted US to laparoscopic US and IOC for the anatomy of the porta hepatis, and accurate measurements of the biliary tree and any ductal stones in choledocholithiasis	Robotic arm assisted	Drop-in L51K Hitachi-Aloka	3–15 MHz	Da Vinci Xi
2023	Otani et al. [19]	Japan	Case Series	3	Rectum	To demonstrate the usefulness of intraoperative sonography (IOUS) for detecting the rectal tumor site in robotic surgery	Robotic arm assisted	Drop-in L43K Hitachi-Aloka	2–12 MHz	Da Vinci Si/Xi
2023	Maertens et al. [20]	UK	Retrospective	32	Rectum	To evaluate the aid of IOUS for safe vessels dissection during complete mesocolic excision	Robotic arm assisted	Drop-in L51K Hitachi-Aloka	3–15 MHz	Da Vinci Xi

## Discussion

In this systematic review, we present a a comprehensive analysis that sheds light on the current state of intraoperative ultrasound for guidance in robotic procedures.

### Summary of main results

Despite the high quality level of evidence supporting laparoscopic ultrasound in various thoraco-abdominal pathologies [30] and the desire to implement RIOUS for over two decades [31], the literature still reflects limited evidence regarding ultrasound guidance during robotic surgery, with relatively small cohort sizes.

All included studies, however, consistently report satisfactory performance of RIOUS. To facilitate the widespread adoption of RIOUS, there is a need for increased adoption of robotic surgical procedures and training for surgeons in IOUS. The utilization of computer assistance for image acquisition and interpretation, through the development and training of machine learning algorithms, could contribute to overcoming operator dependency in ultrasound examinations [32]. In line with the findings of this analysis on RIOUS, image guidance for identifying resection margins by differentiating between healthy and neoplastic tissues has proven particularly useful in oncological diseases [33]. The foremost beneficiary of (R)IOUS thus far is the hepatobiliary field, particularly for the comprehensive anatomical assessment of the biliary and vascular trees [34]. In liver surgery, IOUS plays a well-established role as an intraoperative guidance tool in combination with preoperative CT and MRI imaging. Surgical radicality depends on the detectability of lesions in the different imaging modalities. Techniques such as image fusion of CT/MRI and US, multimodal registration of 2D and 3D imaging modalities as well as (contrast-enhanced) ultrasound contribute to identifying known and preoperatively undetected lesions in order to intraoperatively tailor the surgical strategy [35, 36]. Furthermore, RIOUS was demonstrated to have superior performance compared to conventional LIOUS with a success rate exceeding the one of LIOUS in liver surface exploration (85% vs. 73%, *P* = 0.030) and tool manipulation (79% vs. 57%, *P* = 0.028) [8]. Post-task questionnaires completed by participating surgeons revealed that robotic ultrasound significantly improved probe positioning (80%), reduced fatigue (90%), and was overall more useful than LIOUS (90%) [8]. Facilitating precise probe positioning in RIOUS not only enhances surgical precision but also reduces the physical strain on surgeons during complex procedures [6]. An even more significant benefit is the opportunity to identify otherwise undetected lesions, such as in pancreatic lesions [18]. In benign disease of the biliary tract, IOUS has demonstrated comparable efficacy with intraoperative cholangiography in diagnosing choledocholithiasis, surpassing it in terms of speed and completion rates. This is achieved without the need for a contrast agent, with reduced invasiveness and a decreased risk of infection.The comprehensive assessment of the intra- and extrahepatic biliary tree can be accomplished in an average time of 164.1 s using RIOUS and can be complemented by Doppler ultrasound for assessing the porta hepatis. Precise measurements of the biliary tree and ductal stones enable intraoperative decision-making and management of ductal pathologies, including hybrid approaches [6, 37].

Similarly, rectal tumours were successfully detected using RIOUS, showing its effectiveness in determining the optimal transection line for rectal surgeries, especially in cases where tumours are too high for transanal palpation [16]. Furthermore, in obese patient with rectal cancer RIOUS has been proved to be useful to safely guide vascular dissection [28]. Nephron-sparing surgery, as an alternative to radical nephrectomy, is gaining support as an oncologically equivalent procedure while preserving renal functional capacity [9]. The evolution of robot-assisted partial nephrectomy techniques has ushered in a progressive refinement of tools aiding surgeons in the identification of masses and their vascular networks. A remarkable 100% success rate was demonstrated in identifying kidney lesions with RIOUS [25], optimizing tumour identification, enhancing renal tissue preservation through partial nephrectomy, and ensuring oncological safety [6, 9, 37]. In transoral robotic tongue base resection for obstructive sleep apnoea RIOUS has emerged as an invaluable tool for locating the lingual artery and assessing laryngeal tissues. The integration of RIOUS significantly enhances efficiency by substantially reducing the risk of detrimental intraoperative bleeding complications [29].

Despite the numerous advantages observed across various surgical domains, the integration of intraoperative ultrasound in the robotic field remains underused due to costs consideration, lack of expertise, and the necessity for highly skilled minimally invasive surgeons trained in both robotics and ultrasound techniques [11]. Moreover, although rigid prototypes compatible with robotic arms have been developed [8, 20], they are barely due to cost and the absence of a significant advantages over rigid laparoscopic probes, [6, 38]. In contrast, the adaptability of drop-in probes to all multi- and single-port robotic platforms offers high scalability in clinical applications [6, 8, 20, 38].

### Results in the context of published literature

Applications of IOUS originated in 1960 for the identification of kidney stones in A-mode [34, 39]. Since 1980s, rapid innovations have progressed with applications in hepato-pancreato-biliary and gastrointestinal surgery [40, 41]. In the 1990s, attempts were made to extend the benefits of IOUS to minimally invasive surgery by creating dedicated probes for laparoscopic ultrasound [42]. When used in the robotic setting, these probes were operated by the bedside assistant. However, laparoscopic probes lack the flexibility of IOUS in open surgery (Fig. 2). As robotic platforms do not yet provide integrated ultrasound probes, a specific transducer known as the “drop-in-probe" was recently introduced for robotic surgery. This probe, with a dorsal fin to be grasped with a robotic instrument, can be steered from the console. The small transducer attached to a highly flexible cable, coupled with the motion range of the articulating instrument, facilitates access to anatomical areas that are hard to reach with standard laparoscopic probes.

Furthermore, dedicated robotic console software, such as TilePro (Intuitive Surgical Inc., Sunnyvale, CA, USA), enables the surgeon to create an in-console split-view with side-by-side intraoperative and ultrasound images, or switch between the minimally invasive 3D camera and ultrasound view directly from the console [22]. On platforms with open consoles, surgeons can switch from the integrated robotic display to the external ultrasound screen ideally positioned close to the console surgeon [43]. One of the known limitations of laparoscopy, and even more so in robotic surgery, is the reduced/absent tactile feedback, requiring considerable training is needed to learn to replace haptic with visual information. Consequently, the availability of additional information via RIOUS is particularly relevant in oncology, where achieving zero residual tumour is a major prognostic factor [44–48]. Exploration of the abdominal cavity with LIUOS can detect malignant deposits preventing conversions to open surgery when remaining disease can be excluded [44–47, 49]. Fertility-sparing surgery can be enhanced by IOUS assistance by discriminating healthy from cancerous tissues and to spare ovarian parenchyma [46]. Image-guided organ exploration during surgery could also impact the detection of undiagnosed masses, especially in pancreatic and splenic diseases [11, 18, 19]. Therefore, margin assessment and mapping resection guidance with IOUS are highly relevant in conservative oncologic surgery [50, 51]. However, large-scale future randomized controlled trials (RCTs) are necessary to demonstrate the utility of IOUS in assessing oncological outcomes.

Although the limited number of publications and the presence of heterogeneity among the included studies, mostly consisting of case reports and case series which have been included to report comprehensively the literature evidence, this systematic review on RIOUS procedures highlights the relevance of the technical advances in robotic surgery which underline its expected impact in the field of image-guided surgery.

### Implications for practice and future research

In recent years, an increasing number of robotic platforms has entered the marketplace, a trend expected to persist with decreasing costs and user-friendly platforms for a variety of procedures [3]. However, as the integration of advanced technology based on artificial intelligence and augmented reality is not yet fully automated, making the inclusion of real-time 3D image information into MIS a crucial step in advancing surgical care [10]. Ultrasound-assisted procedures are poised to play a pivotal role in filling this technological gap and are anticipated to grow in parallel with ongoing advancements.

**Fig. 3:**
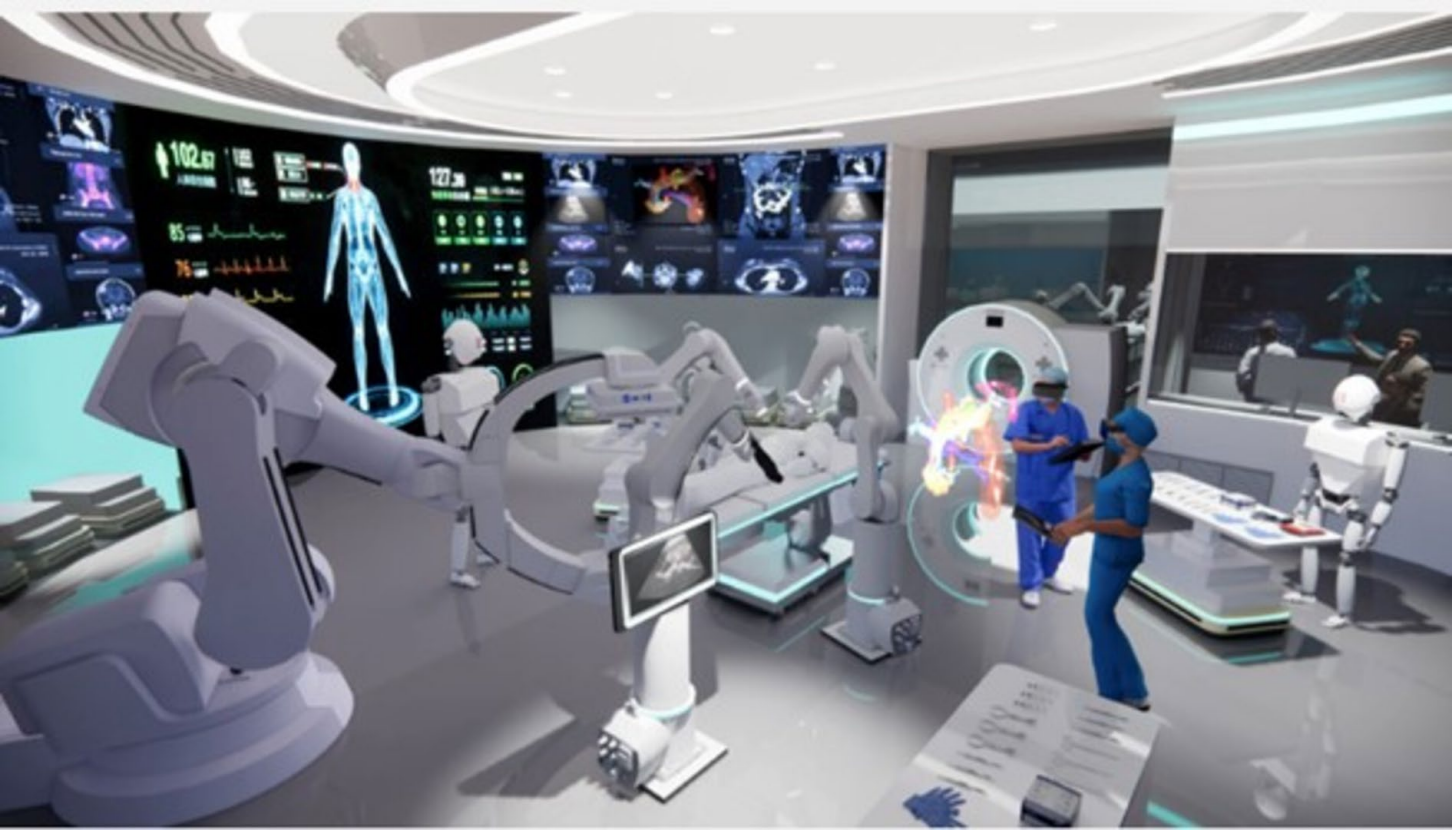
The next-generation hybrid operating room integrating artificial intelligence and robotics for diagnostic imaging, procedure planning and execution: the operating room of the future is envisioned as the centre of a technology ecosystem. Illustrated technology include advanced interactive digital displays with real-time connectivity and AI analytics, mixed-reality environments, and robotic applications for various interventions, imaging (ultrasound, cone-beam CT, intraoperative CT/MRI, etc.), nursing assistance and sterile instrument management, as well as a predictive logistics supply system with Automatic Guided Vehicles [61] (Copyright Barbara Seeliger/ Carlos Amato; Chengyuan Yang; Niloofar Badihi; IHU Strasbourg and Cannon Design USA)

Beyond 3D macroscopic guidance, there is a growing demand for real-time intraoperative tissue analysis, particularly for tailoring the radicality of resection in oncological diseases. In vivo 3D tissue analysis would be ideal for guiding surgery intraoperatively. A variety of intraoperative optical imaging techniques are currently under assessment to complement or potentially replace extemporaneous histopathological analysis [23, 52]. For in vivo tissue, 3D high resolution ultrasound represents a significant step forward in intraoperative analysis within the anatomical context, aiding decision-making on whether resection is required, such as in lymph node metastasis [50]. High (up to 70 MHz) and ultra-high (up to 100 MHz) frequency probes are considered candidates to achieve a resolution of 30 µm, similar to histopathology [53]. An immediate ex vivo imaging system that does not require dedicated sample preparation is full-field optical coherence tomography (FF-OCT), showing a rapid learning curve and analysis of tissue sections similar to [54, 55]. On resected specimens, whole-slide imaging can be used for digital reconstruction as a 3D volume preventing missed lesions for skipped depth slide [56]. In the era of digital surgery, robotic platforms represent computer interfaces capable of integrating multiple modalities of real-time data analysis [10] (Fig. 3). The integration of surgical and imaging sciences will need interdisciplinary training and specific core curricula such as the Master in Image-Guided Surgery, teaching surgeons to perform IOUS, particularly in MIS [57]. Moreover, ongoing studies in deep learning applied to new diagnostic technologies will address the need for standardised IOUS performance and data interpretation by surgeons who may lack adequate radiological expertise [58–60].

## Conclusions

Robotic surgery has become increasingly common in routine clinical practice. Recent technological advancements have paved the way for new tools and equipment in robotic and image-guided surgery. The advantages of robotic instrumentation, including ergonomics, dexterity, and precision of movements, are particularly relevant for robotic intraoperative ultrasound. This systematic review demonstrates the virtue of RIOUS to support intraoperative decision-making and potentially reduce minimally invasive procedure times. Prospective studies, however, are needed to better understand its potential, including disciplines like gynaecologic oncology, where these procedures are not yet commonly performed.

## Data Availability

All data generated or analysed in this review are included in this article and/or its figures. Further enquiries can be directed to the corresponding author.

## References

[1] Gueli Alletti S, Rosati A, Capozzi VA, Pavone M, Gioè A, Cianci S (2022). Use of laparoscopic and laparotomic J-plasma handpiece in gynecological malignancies: results from a pilot study in a tertiary care center. Front Oncol.

[2] Gueli Alletti S, Petrillo M, Vizzielli G, Bottoni C, Nardelli F, Costantini B (2016). Minimally invasive versus standard laparotomic interval debulking surgery in ovarian neoplasm: a single-institution retrospective case-control study. Gynecol Oncol.

[3] Pavone M, Marescaux J, Seeliger B (2023) Current status of robotic abdominopelvic surgery. 秀傳醫學雜誌;(預刊文章):1–15

[4] Pavone M, Seeliger B, Alesi MV, Goglia M, Marescaux J, Scambia G (2023). Initial experience of robotically assisted endometriosis surgery with a novel robotic system: first case series in a tertiary care center. Updates Surg.

[5] Monterossi G, Pedone Anchora L, Gueli Alletti S, Fagotti A, Fanfani F, Scambia G (2022). The first European gynaecological procedure with the new surgical robot HugoTM RAS. A total hysterectomy and salpingo-oophorectomy in a woman affected by BRCA-1 mutation. Facts Views Vis Obgyn.

[6] Glaysher MA, Beable R, Ball C, Carter NC, Knight BC, Pucher PH (2023). Intra-operative ultrasound assessment of the biliary tree during robotic cholecystectomy. J Robot Surg.

[7] Kaczmarek BF, Sukumar S, Petros F, Trinh QD, Mander N, Chen R (2013). Robotic ultrasound probe for tumor identification in robotic partial nephrectomy: initial series and outcomes. Int J Urol.

[8] Schneider CM, Peng PD, Taylor RH, Dachs GW, Hasser CJ, DiMaio SP (2012). Robot-assisted laparoscopic ultrasonography for hepatic surgery. Surgery.

[9] Di Cosmo G, Verzotti E, Silvestri T, Lissiani A, Knez R, Pavan N (2018). Intraoperative ultrasound in robot-assisted partial nephrectomy: state of the art. Arch Ital Urol Androl.

[10] Lecointre L, Verde J, Goffin L, Venkatasamy A, Seeliger B, Lodi M (2022). Robotically assisted augmented reality system for identification of targeted lymph nodes in laparoscopic gynecological surgery: a first step toward the identification of sentinel node. Surg Endosc.

[11] Guerra F, Amore Bonapasta S, Annecchiarico M, Bongiolatti S, Coratti A (2015). Robot-integrated intraoperative ultrasound: initial experience with hepatic malignancies. Minim Invas Ther Allied Technol.

[12] Sokolenko A, Preobrazhenskaya E, Marchetti C, Piermattei A, Zagrebin F, Kuligina E (2023). Origin of residual tumor masses in BRCA1/2-driven ovarian carcinomas treated by neoadjuvant chemotherapy: selection of preexisting BRCA1/2-proficient tumor cells but not the gain of second ORF-restoring mutation. Pathobiology.

[13] Sun Y, Wang W, Zhang Q, Zhao X, Xu L, Guo H (2021). Intraoperative ultrasound: technique and clinical experience in robotic-assisted renal partial nephrectomy for endophytic renal tumors. Int Urol Nephrol.

[14] Moher D, Liberati A, Tetzlaff J, Altman DG, PRISMA Group (2006). Preferred reporting items for systematic reviews and meta-analyses: the PRISMA statement. PLoS Med..

[15] Zhang K, Zhu G, Liu X, Tian J, Gu Y, Zhai M (2019). Robot-assisted laparoscopic retroperitoneal lymph node dissection with concomitant inferior vena cava thrombectomy for metastatic mixed testicular germ cell cancer: a case report. J Med Case Rep.

[16] Otani K, Kiyomatsu T, Ishimaru K, Kataoka A, Hayashi Y, Gohda Y (2023). Usefulness of real-time navigation using intraoperative ultrasonography for rectal cancer resection. Asian J Endosc Surg.

[17] Giulianotti PC, Buchs NC, Coratti A, Sbrana F, Lombardi A, Felicioni L (2011). Robot-assisted treatment of splenic artery aneurysms. Ann Vasc Surg.

[18] Liu Y, Ji WB, Wang HG, Luo Y, Wang XQ, Lv SC (2015). Robotic spleen-preserving laparoscopic distal pancreatectomy: a single-centered Chinese experience. World J Surg Oncol.

[19] Araujo RLC, de Castro LA, Fellipe FEC, Burgardt D, Wohnrath DR (2018). Robotic left lateral sectionectomy as stepwise approach for cirrhotic liver. J Robot Surg.

[20] Di Mitri M, Thomas E, Di Carmine A, Manghi I, Cravano SM, Bisanti C (2023). Intraoperative ultrasound in minimally invasive laparoscopic and robotic pediatric surgery: our experiences and literature review. Children (Basel).

[21] Billings S, Deshmukh N, Kang HJ, Taylor R, Boctor EM (2012) System for robot-assisted real-time laparoscopic ultrasound elastography. In: Medical Imaging 2012: Image-Guided Procedures, Robotic Interventions, and Modeling. SPIE, pp 589–596. 10.1117/12.911086. Accessed 1 Oct 2023

[22] Pessaux P, Diana M, Soler L, Piardi T, Mutter D, Marescaux J (2015). Towards cybernetic surgery: robotic and augmented reality-assisted liver segmentectomy. Langenbecks Arch Surg.

[23] Yakoubi R, Autorino R, Laydner H, Guillotreau J, White MA, Hillyer S (2012). Initial laboratory experience with a novel ultrasound probe for standard and single-port robotic kidney surgery: increasing console surgeon autonomy and minimizing instrument clashing. Int J Med Robot.

[24] Kaczmarek BF, Sukumar S, Kumar RK, Desa N, Jost K, Diaz M (2013). Comparison of robotic and laparoscopic ultrasound probes for robotic partial nephrectomy. J Endourol.

[25] Gunelli R, Fiori M, Salaris C, Salomone U, Urbinati M, Vici A (2016). The role of intraoperative ultrasound in small renal mass robotic enucleation. Arch Ital Urol Androl.

[26] Zhang Y, Ouyang W, Wu B, Pokhrel G, Ding B, Xu H (2020). Robot-assisted partial nephrectomy with a standard laparoscopic ultrasound probe in treating endophytic renal tumor. Asian J Surg.

[27] Zhou Z, Wang Z, Zheng Z, Cao J, Zhang C, He Z (2017). An ‘alternative finger’ in robotic-assisted thoracic surgery: intraoperative ultrasound localization of pulmonary nodules. Med Ultrason.

[28] Maertens V, Stefan S, Mykoniatis I, Siddiqi N, David G, Khan JS (2023). Robotic CME in obese patients: advantage of robotic ultrasound scan for vascular dissection. J Robot Surg.

[29] Chang CC, Wu JL, Hsiao JR, Lin CY (2021). Real-time, intraoperative, ultrasound-assisted transoral robotic surgery for obstructive sleep apnea. Laryngoscope.

[30] Jamal KN, Smith H, Ratnasingham K, Siddiqui MR, McLachlan G, Belgaumkar AP (2016). Meta-analysis of the diagnostic accuracy of laparoscopic ultrasonography and intraoperative cholangiography in detection of common bile duct stones. Ann R Coll Surg Engl.

[31] Angelini L, Papaspyropoulos V (2000). Robotics and telecommunication systems to provide better access to ultrasound expertise in the OR. Minim Invas Ther Allied Technol.

[32] Avesani G, Tran HE, Cammarata G, Botta F, Raimondi S, Russo L (2022). CT-based radiomics and deep learning for BRCA mutation and progression-free survival prediction in ovarian cancer using a multicentric dataset. Cancers (Basel).

[33] Sena G, Paglione D, Gallo G, Goglia M, Osso M, Nardo B (2022). Surgical resection of a recurrent hepatocellular carcinoma with portal vein thrombosis: is it a good treatment option? A case report and systematic review of the literature. J Clin Med.

[34] Schlegel JU, Diggdon P, Cuellar J (1961). The use of ultrasound for localizing renal calculi. J Urol.

[35] Jung EM, Clevert DA (2018). Contrast-enhanced ultrasound (CEUS) and image fusion for procedures of liver interventions. Radiologe.

[36] Torzilli G (2004). Contrast-enhanced intraoperative ultrasonography in surgery for liver tumors. Eur J Radiol.

[37] Dietrich CF, Braden B, Burmeister S, Aabakken L, Arciadacono PG, Bhutani MS (2022). How to perform EUS-guided biliary drainage. Endosc Ultrasound.

[38] Leven J, Burschka D, Kumar R, Zhang G, Blumenkranz S, Dai XD (2005). DaVinci canvas: a telerobotic surgical system with integrated, robot-assisted, laparoscopic ultrasound capability. Med Image Comput Comput Assist Interv.

[39] Knight PR, Newell JA (1963). Operative use of ultrasonics in Cholelithiasis. Lancet.

[40] Sigel B, Coelho JC, Spigos DG, Donahue PE, Renigers SA, Capek V (1980). Real-time ultrasonography during biliary surgery. Radiology.

[41] Fukuda M (1982). Studies on echolaparoscopy. Scan J Gastroenterol.

[42] Jakimowicz JJ, Ruers TJM (2008). Ultrasound-assisted laparoscopic cholecystectomy: preliminary experience. Dig Surg.

[43] Pavone M, Goglia M, Campolo F, Scambia G, Ianieri MM (2023). En-block butterfly excision of posterior compartment deep endometriosis: the first experience with the new surgical robot Hugo™ RAS. Facts Views Vis Obgyn.

[44] De Blasis I, Tortorella L, Macchi C, Arciuolo D, Scambia G, Testa AC (2019). Intraoperative ultrasound diagnosis of metastatic lymph node in serous borderline ovarian tumor. Ultrasound Obstet Gynecol.

[45] Mascilini F, Quagliozzi L, Moro F, Moruzzi MC, Gallotta V, Alletti SG (2018). Role of intraoperative ultrasound to extend the application of minimally invasive surgery for treatment of recurrent gynecologic cancer. J Minim Invas Gynecol.

[46] Mascilini F, Quagliozzi L, Bolomini G, Scambia G, Testa AC, Fagotti A (2019). Intraoperative ultrasound through laparoscopic probe in fertility-sparing surgery for borderline ovarian tumor recurrence. Ultrasound Obstet Gynecol.

[47] Moro F, Uccella S, Testa AC, Scambia G, Fagotti A (2018). Intraoperative ultrasound-guided excision of cardiophrenic lymph nodes in an advanced ovarian cancer patient. Int J Gynecol Cancer.

[48] Marchetti C, Rosati A, De Felice F, Boccia SM, Vertechy L, Pavone M (2021). Optimizing the number of cycles of neoadjuvant chemotherapy in advanced epithelial ovarian carcinoma: a propensity-score matching analysis. Gynecol Oncol.

[49] Jones BP, Saso S, Farren J, El-Bahrawy M, Ghaem-Maghami S, Smith JR (2017). Ultrasound-guided laparoscopic ovarian wedge resection in recurrent serous borderline ovarian tumours. Int J Gynecol Cancer.

[50] Ferrucci M, Milardi F, Passeri D, Mpungu LF, Francavilla A, Cagol M (2023). Intraoperative ultrasound-guided conserving surgery for breast cancer: no more time for blind surgery. Ann Surg Oncol.

[51] Juvekar P, Torio E, Bi WL, Bastos DCDA, Golby AJ, Frisken SF (2023). Mapping resection progress by tool-tip tracking during brain tumor surgery for real-time estimation of residual tumor. Cancers (Basel).

[52] Mascagni P, Padoy N (2021). OR black box and surgical control tower: recording and streaming data and analytics to improve surgical care. J Visc Surg.

[53] Izzetti R, Vitali S, Aringhieri G, Nisi M, Oranges T, Dini V (2021). Ultra-high frequency ultrasound, a promising diagnostic technique: review of the literature and single-center experience. Can Assoc Radiol J.

[54] Yang H, Zhang S, Liu P, Cheng L, Tong F, Liu H (2020). Use of high-resolution full-field optical coherence tomography and dynamic cell imaging for rapid intraoperative diagnosis during breast cancer surgery. Cancer.

[55] Pavone M, Spiridon IA, Lecointre L, Seeliger B, Scambia G, Venkatasamy A (2023). Full-field optical coherence tomography imaging for intraoperative microscopic extemporaneous lymph node assessment. Int J Gynecol Cancer.

[56] Seeliger B, Spiridon IA (2023) Towards optimisation in surgical pathology–the potential of artificial intelligence. BJS Academy. 10.58974/bjss/azbc011

[57] Petter Frühling MD, Seeliger B, Rivera AKU, Freedman J, Giménez M, Digests HPB (2023). Image-guided ablation for liver tumours–an addition to the armamentarium of multidisciplinary oncological and surgical approaches. HPB.

[58] Ho C, Calderon-Delgado M, Chan C, Lin M, Tjiu J, Huang S (2021). Detecting mouse squamous cell carcinoma from submicron full-field optical coherence tomography images by deep learning. J Biophotonics.

[59] Mandache D, Dalimier E, Durkin JR, Boceara C, Olivo-Marin JC, Meas-Yedid V (2018) Basal cell carcinoma detection in full field OCT images using convolutional neural networks. In: 2018 IEEE 15th International Symposium on Biomedical Imaging (ISBI 2018), pp 784–787. https://ieeexplore.ieee.org/abstract/document/8363689

[60] Scholler J, Mandache D, Mathieu MC, Lakhdar AB, Darche M, Monfort T (2023). Automatic diagnosis and classification of breast surgical samples with dynamic full-field OCT and machine learning. J Med Imaging (Bellingham).

[61] Seeliger B, Karagyris A, Mutter D (2023) The role of artificial intelligence in diagnostic medical imaging and next steps for guiding surgical procedures. BJS Academy. https://www.bjsacademy.com/the-role-of-artificial-intelligence-in-diagnostic-medical-imaging-and-next-steps-for-guiding-surgical-procedures. Accessed 17 Nov 2023

